# *Klebsiella pneumoniae* Asparagine tDNAs Are Integration Hotspots for Different Genomic Islands Encoding Microcin E492 Production Determinants and Other Putative Virulence Factors Present in Hypervirulent Strains

**DOI:** 10.3389/fmicb.2016.00849

**Published:** 2016-06-03

**Authors:** Andrés E. Marcoleta, Camilo Berríos-Pastén, Gonzalo Nuñez, Octavio Monasterio, Rosalba Lagos

**Affiliations:** Laboratorio de Biología Estructural y Molecular, Departamento de Biología, Facultad de Ciencias, Universidad de ChileSantiago, Chile

**Keywords:** microcin E492, salmochelin, pathogenicity island, hypervirulent *Klebsiella pneumoniae*, asparagine tRNA gene, liver abscess

## Abstract

Due to the developing of multi-resistant and invasive hypervirulent strains, *Klebsiella pneumoniae* has become one of the most urgent bacterial pathogen threats in the last years. Genomic comparison of a growing number of sequenced isolates has allowed the identification of putative virulence factors, proposed to be acquirable mainly through horizontal gene transfer. In particular, those related with synthesizing the antibacterial peptide microcin E492 (MccE492) and salmochelin siderophores were found to be highly prevalent among hypervirulent strains. The determinants for the production of both molecules were first reported as part of a 13-kbp segment of *K. pneumoniae* RYC492 chromosome, and were cloned and characterized in *E. coli*. However, the genomic context of this segment in *K. pneumoniae* remained uncharacterized. In this work, we provided experimental and bioinformatics evidence indicating that the MccE492 cluster is part of a highly conserved 23-kbp genomic island (GI) named GIE492, that was integrated in a specific asparagine-tRNA gene (asn-tDNA) and was found in a high proportion of isolates from liver abscesses sampled around the world. This element resulted to be unstable and its excision frequency increased after treating bacteria with mitomycin C and upon the overexpression of the island-encoded integrase. Besides the MccE492 genetic cluster, it invariably included an integrase-coding gene, at least seven protein-coding genes of unknown function, and a putative transfer origin that possibly allows this GI to be mobilized through conjugation. In addition, we analyzed the asn-tDNA loci of all the available *K. pneumoniae* assembled chromosomes to evaluate them as GI-integration sites. Remarkably, 73% of the strains harbored at least one GI integrated in one of the four asn-tDNA present in this species, confirming them as integration hotspots. Each of these tDNAs was occupied with different frequencies, although they were 100% identical. Also, we identified a total of 47 asn-tDNA-associated GIs that were classified into 12 groups of homology differing in theencoded functionalities but sharing with GIE492 a conserved recombination module and potentially its mobility features. Most of these GIs encoded factors with proven or potential role in pathogenesis, constituting a major reservoir of virulence factors in this species.

## Introduction

*Klebsiella pneumoniae* is a Gram-negative bacterium normally considered as an opportunistic pathogen causative of nosocomial infections (Podschun and Ullmann, 1998; Brisse et al., 2006). Although it is commonly carried asymptomatically in different tissues of healthy individuals, it can also cause a variety of mild to severe infections involving among others the urinary tract, lungs, abdominal cavity, and intravascular catheters (Ko et al., 2002; Shon et al., 2013). However, in the last two decades an increasing number of community-acquired invasive *K. pneumoniae* infections have emerged, mainly in the form of pyogenic liver abscesses that are often accompanied by severe metastatic infections such as endophthalmitis, meningitis, and necrotizing fasciitis (Chang and Chou, 1995; Siu et al., 2012; Struve et al., 2015). Initially, those cases were restricted to Southeast Asia but currently an increasing number of cases are being reported globally. Hence, this agent is now considered as an urgent threat to public health, not only by the hypervirulent strains associated with severe infections but also because of the emergence of multidrug-resistant strains associated with hospital outbreaks (Munoz-Price et al., 2013; Holt et al., 2015). The increasing prevalence of multiresistance and hypervirulence determinants among *K. pneumoniae* isolates have attracted the attention of several research groups and motivated the whole-genome sequencing of a rapidly growing number of strains, as a mean of deciphering the molecular mechanisms underlying these traits (Holt et al., 2015; Struve et al., 2015). Genomic analysis of the hypervirulent strains revealed that their virulence is mainly determined by a specific gene profile acquired horizontally (Holt et al., 2015).

The acquisition of virulence and resistance determinants through horizontal gene transfer among bacteria is commonly mediated by a plethora of genetic mobile elements such as plasmids, bacteriophages, transposons and GIs. In particular, the latter elements have been described as a significant source of virulence-associated genes in *K. pneumoniae* and other species from *Enterobacteriaceae* (Dobrindt et al., 2004; Gal-Mor and Finlay, 2006). GIs are discrete chromosomal DNA segments differing between closely related bacterial strains to which usually some past or present mobility is attributed. Different GIs have the following common features: (1) their size typically ranges from 10 to 200 kbp; (2) they show a GC content and codon usage that differs from the chromosome average; (3) they are often inserted at tRNA genes (tDNAs); (4) they are flanked by direct repeats that arise upon the integration event, that corresponds to the 3′ portion of the tDNA; (5) they usually harbor integrase-coding genes that catalyze the site-specific recombination event leading to the island integration or excision; (6) some of them carry other mobility related genes encoding transposases or factors that allow the conjugal transfer of the element; and (7) they frequently carry genes conferring new metabolic capabilities to their hosts (Dobrindt et al., 2004; Boyd et al., 2009; Juhas et al., 2009). GIs coding for virulence-related determinants are known as PAIs. In *K. pneumoniae*, several virulence and mobility factors are encoded in PAIs, including fimbrial assembly proteins, yersiniabactin siderophore synthesis and uptake determinants, colibactin toxin synthesis proteins, a virulence-related phospholipase D, and conjugative transfer-related proteins (Koczura and Kaznowski, 2003; Lin et al., 2008; van Aartsen, 2008; Putze et al., 2009; Chen et al., 2010; Lery et al., 2014). Among other putative virulence-related factors of *K. pneumoniae* likely encoded in PAIs are the determinants required for MccE492 and salmochelin siderophore production (Marcoleta et al., 2013a; Lai et al., 2014).

Microcin E492 is an 8-kDa pore-forming bacteriocin produced by *Kp*RYC492, which has antibacterial activity against members of the family *Enterobacteriaceae* (de Lorenzo, 1984). For bactericidal activity, MccE492 requires the posttranslational attachment to its C-terminus of enterobactin glycosylated-derivatives denominated salmochelins, which are the moiety recognized by the target cell receptors that mediate toxin uptake (Lagos et al., 2009). Both enterobactin and salmochelins function as siderophores, allowing bacteria to scavenge iron from the extracellular milieu. The combined production of MccE492 and salmochelins under iron-limiting conditions is thought to be a strategy to defend the salmochelin utilization system against competitors for this iron source. This kind of molecules has advantages over enterobactin as siderophores; namely, the glycosylation allows the evasion against the mammalian catecholate siderophore-binding protein siderocalin (Fischbach et al., 2006). Moreover, they are produced in larger amounts than enterobactin in *Salmonella* Typhimurium (Hantke et al., 2003; Müller et al., 2009). Although no direct involvement of MccE492 in pathogenesis has been demonstrated so far, two recent large-scale genomic comparisons among different *K. pneumoniae* isolates revealed a high prevalence of this factor along with the salmochelin synthesis determinants in the genome of hv*Kp* strains. This strongly suggests that they may play a role as virulence factors in this species probably as a tool in the competition with the host microbiota during colonization (Holt et al., 2015; Struve et al., 2015).

The genetic determinants required for MccE492 production were originally described as part of a ∼13-kbp cluster located in the chromosome of *Kp*RYC492, that was cloned and characterized in *E. coli* (Wilkens et al., 1997; Lagos et al., 2001). This 13-kbp segment showed to be sufficient to allow the heterologous production and export of MccE492 with the same biochemical properties than the MccE492 purified from *Kp*RYC492. However, the genomic context of the MccE492 production cluster in its former host remained unexplored until the recent sequencing, assembly, and annotation of the *Kp*RYC492 genome, where a preliminary inspection indicated that the MccE492 cluster could be located inside a GI (Marcoleta et al., 2013a). In this work, we performed bioinformatics and experimental analyses indicating that the MccE492 cluster actually forms part of an unstable 23-kbp GI (named GIE492) with hallmark features of this kind of genetic elements. The mobility properties of this island were studied, as well as its structure, prevalence and conservation among a previously sequenced set of *K. pneumoniae* clinical isolates. In addition, we performed a comprehensive analysis of the asn-tDNA loci of this species, identifying and classifying a total of 47 GIs integrated at these genes, and providing new insights about their properties as GIs integration hotspots.

## Materials and Methods

### Bacterial Strain and Plasmids

Bacterial strain and plasmids used in this work are shown in **Table 1**.

**Table 1:** Bacterial strain and plasmids used in this work.

Bacterial strain or plasmid	Relevant genotype or features	Source
***K. pneumoniae* strain**		
RYC492	Microcin E492 producing strain, isolated from a stool sample. Kan^r^.	Asensio et al., 1976
**Plasmids**		
pCA24N	(ASKA-). Cam^r^	Kitagawa et al., 2005
p*int*	pCA24N-derivative. Allows the IPTG-inducible expression of the integrase gene of GIE492 in *Kp*RYC492.	This study
pUC57	pUC19-derived cloning vector. Amp^r^	Genscript

### Growth Conditions

Bacterial growth was performed incubating with shaking (180–220 rpm) at 37°C in Luria Broth during the indicated time. As required, culture medium was supplemented with mitomycin C (Sigma) at the indicated concentrations, and/or IPTG 1 mM and antibiotics. Antibiotics were used in the following concentrations: kanamycin (Kan) 50 μg/ml and chloramphenicol (Cam) 100 μg/ml.

### Cloning and Recombinant DNA Techniques

Conventional procedures not further detailed herein, such as plasmid and genomic DNA isolation, agarose-gel electrophoresis, restriction enzyme digestion, ligation, transformation, and conventional PCR were performed according standardized methods (Green and Sambrook, 2012) and guidelines from reagent’s manufacturers.

### Oligonucleotide Primers

The primers used for conventional, nested, and quantitative PCR are listed in Supplementary Table 1.

### RNA Extraction and cDNA Synthesis

RNA extraction was performed starting from *Kp*RYC492 cells pelleted from 3 to 10 ml culture. Cell pellet was suspended in 1 mL of TRIzol (Invitrogen) and 500 μl of 0.1 mm acid-washed glass beads (Sigma) were added. The mixture was vortex-shaken vigorously in four steps of 2 min, incubating 1 min in ice between each step. Then, 1 ml of absolute ethanol (Merck) was added mixing well. The resulting mixture was then loaded into a Direct-zol RNA Miniprep kit column (Zymo Research) and the procedure continued until obtaining the isolated RNA dissolved in RNase-free water, following the guidelines of the kit’s manufacturer. The resulting RNA was quantitated in an Epoch microplate spectrophotometer (BioTek), and its purity was evaluated through the measurement of the ratio between absorbance at 260 and 280 nm. Ratios between 1.85 and 2.0 were typically obtained. The integrity of the purified RNA was further evaluated by agarose-gel electrophoresis, corroborating the presence of two defined bands corresponding to non-degraded 16S and 23S ribosomal RNAs.

cDNA synthesis was performed starting from 2 μg of total RNA using the Maxima First Strand cDNA Synthesis Kit for RT-qPCR with dsDNase (Thermo Scientific), following the manufacturer’s guidelines. Five-minute treatment with dsDNase was found to be necessary to efficiently eliminate contaminant genomic DNA.

### Quantitative PCR (qPCR) Measurements

Quantitative PCR assays starting from genomic DNA (excision frequency determinations) or retro-transcribed RNA (cDNA, gene expression measurements) were performed using a Stratagene Mx3005P real-time PCR platform and the amplification reagent mixture SensiMix^TM^ SYBR Hi-ROX (Bioline), in a final volume of 20 μl. qPCR data was obtained and analyzed with the MxPro software. For each primer pair, efficiency of amplification was calculated from the slope of standard curves constructed ploting the threshold cycle (*C*_t_) versus the logarithm of initial DNA concentration, using 10-fold serially diluted template samples, as described previously (Rujiter et al., 2009). Standard curves were made starting with genomic DNA samples (excision frequency measurements) or cDNA samples (gene expression measurements). The amplification profile used consisted of an initial denaturation step of 15 min at 95°C, 40 cycles of 15 s at 95°C, 15 s at 56°C, and 15 s at 72°C; a final denaturation curve check was done to assure the occurrence of a single melting temperature of the amplified DNA.

For excision frequency determinations two sets of primers were used. P3 and P5 were used to quantify the copy number of the chromosomal scar left after GIE492 excision. rpoD_realFw and rpoD_realRv were used to quantitate the total chromosome number in each genomic DNA sample, as they amplify *rpoD* gene that is present in a single copy in the *Kp*RYC492 genome. Excision frequency was determined as the quotient of the scar copy number and total chromosome copy number, as described previously (Quiroz et al., 2011).

For gene expression measurements, 1 μl of the synthesized cDNA was used in a final reaction volume of 20 μl. For each condition measured, a “no RT” control was performed starting from an equivalent amount of RNA treated with the dsDNase of the cDNA synthesis kit, but not subjected to reverse-transcription (adding water to complete the 20 μl final volume). Relative expression values were obtained calculating the target transcript copy number normalized by the copy number of a reference gene, considering primer efficiencies (E) of both primer sets by using the formula (1 + *E*_target_)^C_t target_^/(1 + *E*_reference_)^C_t reference_^. As reference genes, we tested three commonly used in bacterial gene expression measurements: *gapA* (coding for glyceraldehyde-3-phosphate dehydrogenase), *rpoD* (coding for RNA polymerase sigma subunit) and the gene coding for 23S rRNA. From these three, *rpoD* showed the most constant expression when comparing samples from different growth phases. Consequently, *rpoD* was used in those assays.

### p*int* Construction

To achieve the inducible overexpression of the GIE492 integrase-coding gene in *Kp*RYC492, the p*int* plasmid was constructed. To this end, the coding region of the GIE492 integrase gene flanked by *Not*I (5′ end) and *Pst*I (3′ end) was synthesized and cloned in pUC57. Then, the *Not*I-*Pst*I fragment was subcloned in the expression vector pCA24N (Kitagawa et al., 2005) using these restriction sites, resulting in p*int*. To validate the suitability of this expression system in *Kp*RYC492, we corroborated that pCA24N allows the IPTG-inducible expression of GFP, detecting this protein by immunoblot using an anti-GFP antibody. Also, green fluorescence of the induced cells was confirmed by visual inspection and fluorescence microscopy (data not shown).

### Bioinformatics Analyses and Source of DNA Sequences

Short reads from *K. pneumoniae* isolates used for mappings of GIE492 (previously described in Struve et al., 2015) were obtained from the European Nucleotide Archive and are listed in Supplementary Table 2. Assembled genome sequences used in this study, except for *Kp*RYC492 were obtained from NCBI Genome database^1^ . Genome inspection, sequence analysis and multiple alignments were performed using the platforms Artemis (Rutherford et al., 2000), ProgressiveMauve (Darling et al., 2010) and NCBI BLAST. Alignment visualization and formatting was performed using Jalview 2 software (Waterhouse et al., 2009). Short read mapping to reference sequences and *de novo* assembly were performed using the platform UGENE (Okonechnikov et al., 2012). Initial identification of GIs among *K. pneumoniae* strains was performed using the MobilomeFinder web platform (Ou et al., 2007).

### Statistical Analysis

Statistical analyses were performed using the Prism 6 platform. One-way and two-way ANOVA tests were used to compare single or multiple datasets, respectively. Bonferroni’s multiple comparisons post-test was used to determine the significance between pairs of samples from different conditions.

## Results

### The Microcin E492-Production Gene Cluster Is Located in a Genomic Context Harboring Hallmark Features of Genomic Islands

In a previous work, we reported the whole-genome sequencing of the strain *Kp*RYC492 (Marcoleta et al., 2013a) from which the antibacterial peptide MccE492 was first isolated (de Lorenzo, 1984). The obtained short reads were assembled using a multi-reference iterative-mapping approach, generating a 5,095,761-Mbp open chromosome (GenBank accession number APGM01000001.1). The resulting assembled sequence was annotated using the NCBI Prokaryotic Annotation Pipeline, and then manually curated.

**FIGURE 1:**
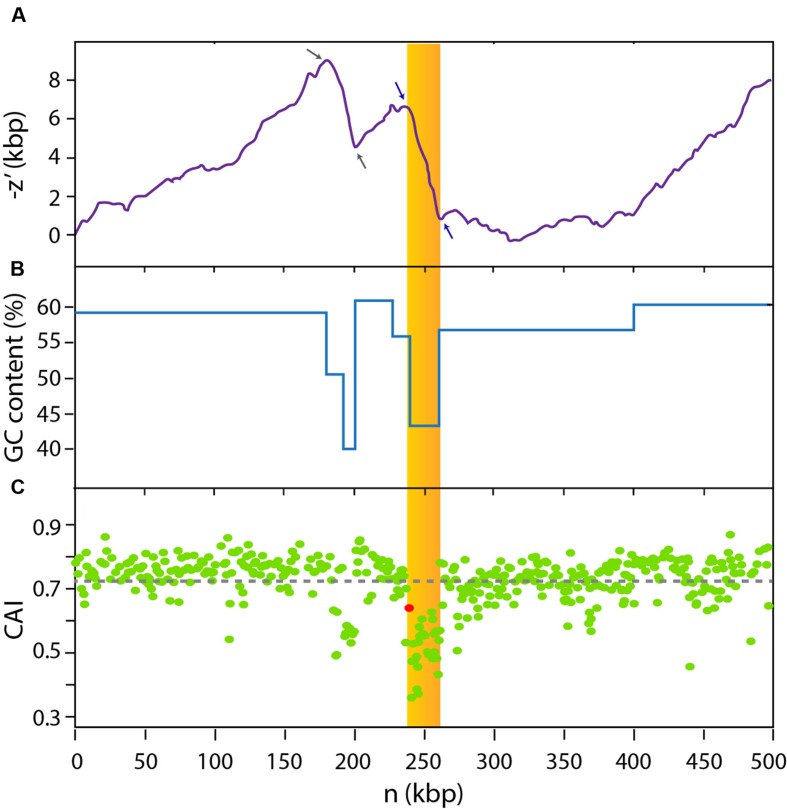
**The MccE492-production gene cluster is located inside a 23-kbp genomic region with biased GC content and codon usage**. A 500-kbp region of the *Kp*RYC492 chromosome (coordinates 1,466,268–1,966,267) was analyzed (*n*: distance in base-pairs to the start of the segment). GC profile algorithm calculations **(A)** indicated that two segmentation points (blue arrows) delimitate a 23-kbp region (dark yellow area) comprising MccE492 production cluster. This region showed an average GC content of 44% **(B)**. Codon adaptation index (CAI) calculations revealed that genes located inside this region also show a strong bias in codon usage **(C)**. The red dot corresponds to the CAI value for the integrase gene located between the MccE492 coding gene and the asn-tRNA gene. Gray arrows in **(A)** indicate two additional segmentation points delimitating a further region unrelated to GIE492 that could also be acquired horizontally.

In order to gain information about the genomic context of the MccE492 gene cluster, we examined the sequence of the *Kp*RYC492 chromosome and noticed that it is located inside a region with hallmark features of GIs. First, roughly 300-bp downstream of the *mceA* gene (coding for the MccE492 structural protein) there is a gene encoding a tyrosine recombinase, a family of proteins that commonly catalyzes the excision and integration of such kind of mobile elements (Boyd et al., 2009). Second, immediately adjacent to the integrase gene, there is a gene encoding an asn-tRNA that could be the integration site of the putative GI, as reported for several GIs from *Enterobacteriaceae* (Schmidt and Hensel, 2004; Schubert et al., 2004; Lin et al., 2008). Additionally, the analysis of the 500-kbp region of the chromosome comprising the MccE492 gene cluster and its surroundings (coordinates 1,466,268–1,966,267) revealed GC-content and codon usage bias (**Figure 1**). This region was analyzed using the GC-Profile tool, which calculates the negative cumulative GC profile (-z′) (Gao and Zhang, 2006). This index is proportional to the expression (C*_n_* + G*_n_*) – (A*_n_* + T*_n_*), where A*_n_*, C*_n_*, G*_n_* and T*_n_* are the cumulative numbers of the bases A, C, G, and T, respectively, occurring from the first to the *n*th base in the DNA sequence inspected. Hence, for a GC-poor DNA stretch, -z′ is approximately a monotonously decreasing linear function of *n*. Zones in the -z′ curve with an abrupt change of slope are named segmentation points, and correspond to the limits of regions with a G+C content distinct from the contiguous segments (Gao and Zhang, 2006). The search for segmentation points has probed to be an effective way to identify putative bacterial GIs (Zhang and Zhang, 2004). This way, we identified two segmentation points (blue arrows, **Figure 1A**) delimiting the 21-kbp region comprised between coordinates 1,706,490 and 1,727,482 of the *Kp*RYC492 chromosome (showed as a yellow area). This region, which includes the 13-kbp MccE492 gene cluster, presented a ∼44% GC content (**Figure 1B**), significantly lower than the calculated chromosome average (57.9%). The 500-kbp region was further analyzed in order to detect codon usage bias as an additional evidence for the presence of a horizontally acquired mobile element. To this end, we used the CAIcal tool (Puigbo et al., 2008) to calculate the codon adaptation index value (CAI) for each coding region present in the 500-kbp segment (a total of 421 open reading frames). CAI value is a measure of how well adapted is each protein coding gene to the codon usage preferences of the host organism (Sharp and Wen-Hsiung, 1987), calculated from the usage frequency of each codon of a particular gene compared with the usage frequency of a reference set of genes. It can range from 0 to 1, where 1 indicates that the analyzed coding region is constituted only by the host’s preferred codon for each amino acid, while a low value indicates poor adaptation. In this case, we used as reference the codon usage table of *K. pneumoniae* available in the Codon Usage Database^2^. CAI values for each coding region over the 500-kbp chromosome segment explored were plotted in **Figure 1C**. The region showing a distinct GC content mentioned above also presented codon usage bias, with a large group of genes having a codon usage poorly adapted to *K. pneumoniae* preferences (low CAI values). The coding region situated in the left limit of the region (red dot) corresponded to the integrase gene of the putative GI, located immediately downstream of the asn-tDNA. Among the genes with the lowest CAI values were those coding for MccE492 and its immunity protein (0.36 and 0.47, respectively). Inspection of the upstream region also showing GC content and codon usage bias (delimitated by gray arrows in **Figure 1A**) indicated that it encodes several glycosyltransferases putatively related with the capsular polysaccharide synthesis. This segment was found to be present only in some strains of *K. pneumoniae* and thus likely acquired by horizontal transfer.

**FIGURE 2:**
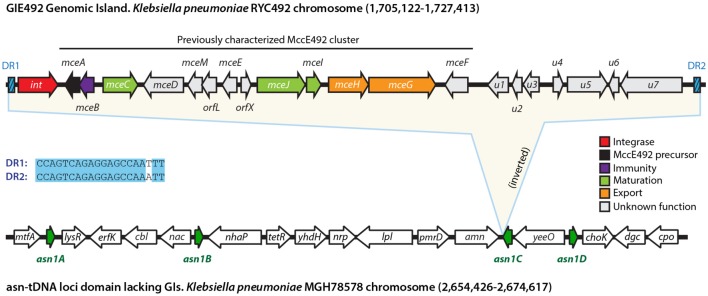
**GIE492 features and genomic context**. GIE492 is a 22,291-bp DNA segment flanked by direct repeats (DR1 and DR2). It harbors an integrase-coding gene, the previously characterized MccE492-production gene cluster, and at least seven additional protein-coding genes of unknown function, provisionally designed *u1* to *u7*. This GI is inserted in one of the four copies of the asn-tDNA present in *K. pneumoniae* (*asn1A* to *asn1D*). These *asn1* loci cluster together in a 20-kbp domain with a gene organization conserved in all the strains that do not harbor *asn1*-GIs inserted, as the case of the chromosome of *K. pneumoniae* MGH78578 reference strain. In this scheme, we show the *asn1* domain of MGH78578 and particularly *asn1C*, where GIE492 is integrated in the equivalent region of *Kp*RYC492 chromosome.

To further delimitate the putative GI comprising the MccE492 gene cluster (from now referred as “GIE492”), we performed multiple sequence alignments comparing the genomic context of this island with the equivalent genomic region of several *K. pneumoniae* strains. Additionally, we used BLASTn to search for the presence of direct repeats expected to delimitate the island. From these analysis and the information described above, we concluded that GIE492 is a 22,3-kpb island located between coordinates 1,705,122 and 1,727,413 of the *Kp*RYC492 genome, which is flanked by a 17 (perfect) to 20-bp (imperfect) direct repeat that includes the last 16 bases of the asn-tDNA (**Figure 2**). The equivalent region of the *K. pneumoniae* MGH78578 reference strain, which does not harbor GIs, is also depicted (coordinates 2,654,426–2,674,617) and the place in which GIE492 insertion occurred in RYC492 genome is marked as *asn1C*. This ∼20-kbp segment of the MGH78578 chromosome comprises all of the four copies of the asn-tDNA present in its genome, which are 100% identical and code for asn-tRNA with GUU anticodon. Since no systematic nomenclature for *K. pneumoniae* tRNA genes has been established, for convenience those loci were denominated *asn1A* to *asn1D*. The shared number indicates that all of them encode a tRNA with the same anticodon and the different letters distinguish each copy by its particular gene context.

Besides the previously described ∼13-kbp MccE492 cluster (encompassing genes *mceA* to *mceF*), GIE492 comprised the integrase-coding gene and at least seven putative genes of unknown function (provisionally designated *u1* to *u7*). The search for homologs with known function using BLAST indicated that *u1* encodes a putative 162-amino acids protein with significant identity to type-11 methyltransferases, which transfer methyl groups from *S*-adenosyl methionine (SAM) to DNA, RNA, proteins, and also to small molecules such as catechols (Schubert et al., 2003; Nelson et al., 2007). *u5* encodes a putative 352-amino acids protein with tetratricopeptide repeats (TPR), which are proposed to participate in protein–protein interactions and scaffolding of higher order macromolecular complexes. *u6* encodes a putative 626-amino acids protein with an NTPase motif and a topoisomerase-primase nucleotidyl transferase/hydrolase domain, related to endonuclease proteins of the old-like family (overcoming lysogenization defect). On the other hand, *u2*, *u3*, *u4*, and *u6* encode hypothetical proteins of 73, 120, 57, and 53 amino acid residues, respectively, that are conserved among *K. pneumoniae* but have no identified or proposed function. Because of the scant information obtained after searching the databases for characterized homologs, at this point we were not able to infer if these uncharacterized genes play a role in MccE492 synthesis.

As a means to evaluate the presence and conservation of GIE492 among other *K. pneumoniae* sequenced isolates, we searched public databases using BLAST tool and noticed that besides RYC492, there were two additional strains with assembled chromosome that harbored the MccE492 production determinants, namely strains 1084 and RJF999 (accessions CP003785.1 and CP014010.1, respectively). Additionally, in a recent study by Struve et al. (2015) 69 clinical isolates from different geographic locations were sequenced reporting that many of them encoded MccE492 and salmochelin gene determinants. However, no details were provided regarding if all the genes required for synthesizing those molecules were present, nor the genomic context in which they would be placed. Since only the raw reads from the genomes of those isolates were publicly available, a previous assembly was required for their analysis. To this end, we used a 100-kbp region from the RYC492 chromosome comprising GIE492 and its surroundings as a reference to map the reads from each of the isolates, obtaining a partial assembly of this region. After inspecting the genomes of strains 1084, RJF999 and the isolates described by Struve et al. (2015), we observed that in all the chromosomes where MccE492 determinants were found (a total of 35), they were part of the GIE492 island with the same structure described for RYC492 (**Figure 2**). Moreover, they shared an overall 99% sequence identity, were inserted in the same asn-tDNA locus (*asn1C*), and had identical direct repeats. Regarding the clinical samples analyzed, a total of 33 out of 69 isolates (48%) included the GIE492 (Supplementary Table 2). From these, six isolates were previously reported as lacking MccE492 and salmochelin production determinants (A5054, CAS692, CAS905, CAS906, Sp29, and Sp221), but now we confirmed that they actually carry the whole island. Regarding the phylogenetic relationship among the GIE492-positive isolates, 31 of them and also strain 1084 belong to a single clonal complex denominated CC23 or to a closely related group (Struve et al., 2015). Moreover, after performing genomic BLAST we found a close relationship between 1084, RJF999, and RYC492, indicating that the last two would also belong to this complex. By contrast, Sp29 and Sp221 were very distant from CC23, suggesting that in these isolates GIE492 was independently acquired. Remarkably, 24 out of 26 isolates from liver abscesses carry GIE492 including samples collected between 1996 and 2012 in North America, Europe, Asia, and Africa. This correlation suggests that MccE492 and or salmochelin production may be important virulence factors participating in the development of this kind of aggressive infection.

### GIE492 Is an Unstable Genomic Island

**FIGURE 3:**
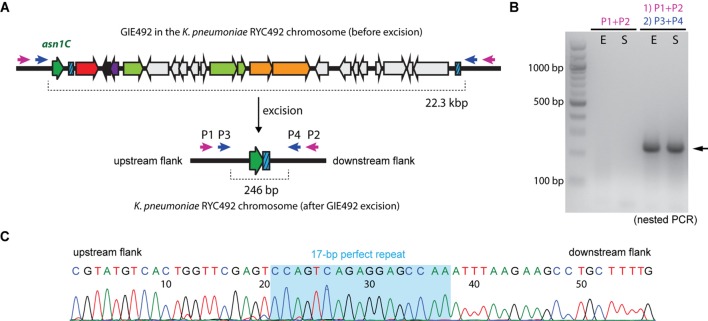
**Detection of GIE492 excision**. **(A)** Schematic representation of the excision event. Direct repeats are depicted as blue boxes. Two primer sets (P1/P2, purple arrows; and P3/P4, blue arrows) were designed to hybridize the regions outside and next to the island borders. Before excision, P3 and P4 delimitate a region of more than 22 kbp. After excision, these primers become 246-pb apart from each other, delimitating a region (scar) susceptible to be amplified by PCR. **(B)** PCR-amplification of the scar using genomic DNA of *Kp*RYC492 extracted in exponential (E) or stationary (S) phase of growth and primers P1 to P4. A first amplification with P1+P2 and a subsequent amplification with P3+P4 (nested PCR) were required to detect the scar, visualized as a prominent band of the expected size (black arrow). **(C)** Partial nucleotide sequence of the bands obtained in **(B)** confirming that they correspond to the scar left after GIE492 excision, including the 17-bp perfect repeat (shaded in light blue).

The presence of the direct repeats and a gene encoding an apparently functional integrase inside this GI strongly suggests that it is unstable, i.e., able to excise itself from the chromosome under certain conditions. To test this hypothesis we conducted a conventional PCR strategy assay to detect the excision event using oligonucleotide primers designed to hybridize the zones immediately adjacent to the island borders (P1–P4; **Figure 3A**). P1 and P2 are located more than 22-kbp away from each other when GIE492 is integrated into the chromosome, so no amplification should be observed after performing a PCR reaction. In contrast, after island excision the primers become close enough to generate a ∼300-bp amplicon. This way, we searched for such amplicon performing PCR reactions using primers P1 and P2 on genomic DNA extracted from *Kp*RYC492 at exponential or stationary phase of growth in LB medium (**Figure 3B**). No amplicons of the expected size were obtained after several attempts, probably due to the low frequency of the excision event. To increase the sensitivity of the detection we used nested PCR, where a second primer pair (P3 and P4) was designed to amplify a region internal to the amplicon generated in the previous PCR reaction using P1 and P2. With this approach we successfully obtained an amplicon of the expected size (246 bp; **Figure 3B**, black arrowhead) that was observed both in exponential and stationary phase. To corroborate this result, we purified and sequenced the gel bands (**Figure 3C**). The sequence obtained included the 17-bp perfect repeat detected by bioinformatics analysis and upstream and downstream regions corresponding to the zones of the chromosome adjacent to GIE492.

It is generally accepted that upon excision, GIs form a circular intermediate unable to replicate, which in some cases could be detected. We attempted to detect such circular intermediate using a similar nested PCR approach and PCR primers that become convergent upon GI circularization, but no amplification was observed in all the conditions tested.

### GIE492 Excision Frequency Increased in Presence of Mitomycin C or upon Overexpression of the Island-Encoded Integrase

**FIGURE 4:**
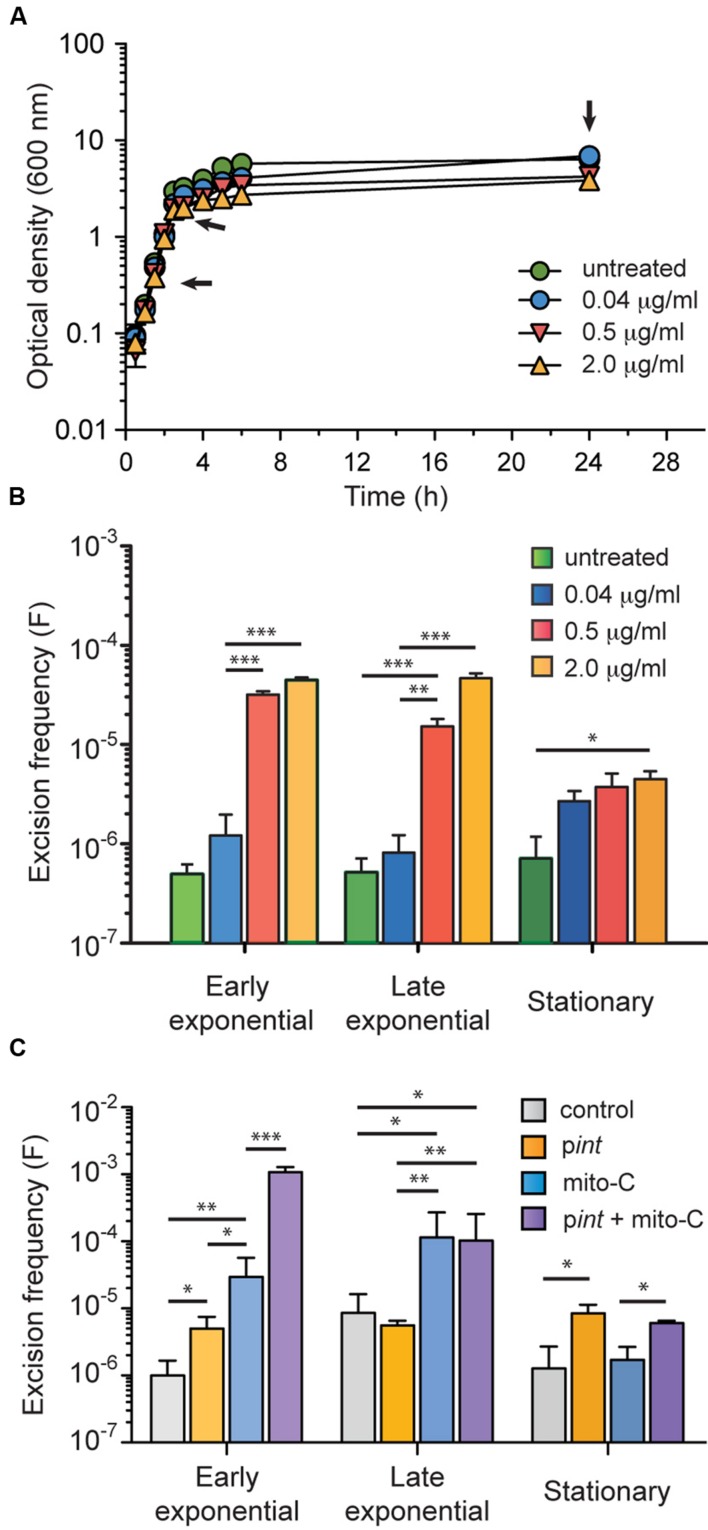
**GIE492 excision frequency increased upon addition of mitomycin C to the culture medium or overexpressing the island-encoded integrase**. Excision frequency was measured by qPCR starting from genomic DNA isolated from *Kp*RYC492 cells cultured until different phases of growth in a medium without or supplemented with mitomycin C (mito C). **(A)** Growth curves of *Kp*RYC492 in LB medium supplemented with up to 2 μg/ml mito C. Arrows indicate the points from which genomic DNA was extracted. **(B)** Dose-dependent mito C-mediated increase of GIE492 excision frequency. **(C)** Effect of overexpressing the island-encoded integrase over the excision frequency. *Kp*RYC492 cells were transformed with either p*int* (allowing IPTG-inducible expression of *int* gene) or pCA24N (control) and grown in LB supplemented with IPTG, or IPTG plus mito-C until the indicated phase of growth. Error bars correspond to the standard deviation of two measurements from three independent experiments. ^∗^*p* < 0.05, ^∗∗^*p* < 0.01, ^∗∗∗^*p* < 0.001.

In order to determine the excision frequency under conventional growth conditions, we adapted a qPCR-based strategy previously described by Quiroz et al. (2011). Oligonucleotide primers hybridizing regions adjacent to GIE492 integration site were used to determine the copy number of the chromosomal scar left after island excision (i.e., the number of chromosomes that lost the island). A second primer pair was designed to amplify the *rpoD* gene, which is in a single copy in the *Kp*RYC492 genome, accounting for the total chromosome number present in each sample. From this, the excision frequency was estimated as the copy number of scars (left after GI excision) divided by the total chromosome copy number. Genomic DNA was extracted from *Kp*RYC492 cells in early exponential, late exponential and stationary phase of growth, and the excision frequency was determined (**Figures 4A,B**, untreated series). The estimated GIE492 excision frequency for *Kp*RYC492 cells grown in LB medium was around 6 × 10^-7^ in the three cases, which falls in the range of frequencies calculated for other enterobacterial GIs (Middendorf et al., 2004).

Previous reports indicate that excision of GIs and prophages can be induced through the addition of the DNA-damaging antibiotic mitomycin C (Bellanger et al., 2008; Ahmed et al., 2012; Puymège et al., 2013). To test if GIE492 follows this behavior, we determined the excision frequency during the growth curve in LB medium supplemented with increasing amounts of mitomycin C (**Figures 4A,B**). The range of concentrations selected did not cause any severe effect over cell growth. As expected, a dose-dependent increase of the excision frequency was observed in presence of mitomycin C, especially in early and late exponential phases of growth, reaching up a 100-fold increment.

To test the participation of the GIE492-encoded integrase in the excision process we examined the effect of overexpressing its coding gene (*int*). To this purpose, *int* was cloned in the IPTG-inducible expression vector pCA24N generating p*int*. Then, we transformed *Kp*RYC492 cells with p*int* or with pCA24N (control) and compared the excision frequency at different stages of cell growth in presence of 1 mM IPTG. Additionally, we tested the combined effect of integrase overexpression and mitomycin C addition. As shown in **Figure 4C**, integrase overexpression caused a moderate fourfold increase in the excision frequency that was significant only in early exponential and stationary phases (control vs. p*int*). This effect is lower than the excision induction caused by mitomycin C (control vs. mito C). However, overexpression of *int* gene in presence of mitomycin C resulted in a synergistic increase of the excision frequency that was noticed mainly in the early exponential phase of growth. These results suggest that the GIE492 *int* gene is involved in the excision of this island, and that the mitomycin C-mediated induction of this process probably occurs through a different mechanism than the induction of the integrase gene expression. We also tried to study the consequences of the deletion of *int* gene on the excision of GIE492. However, after many attempts to delete this or other genes using different methods no *Kp*RYC492 mutants were obtained.

### GIE492 Gene Expression

**FIGURE 5:**
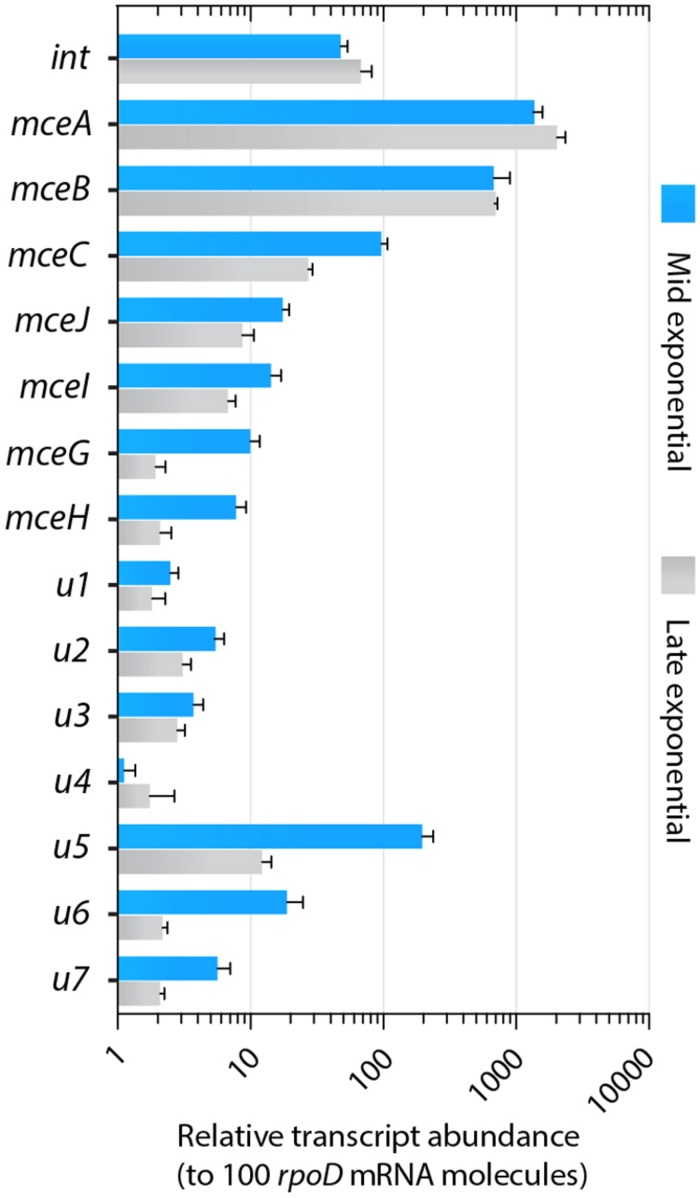
**Genes from GIE492 encoded in the MccE492-production cluster and genes unrelated to this bacteriocin production are transcribed at comparable levels**. Relative abundances of GIE492-encoded transcripts were measured by qRT-PCR, starting from total RNA isolated from *Kp*RYC492 cells cultured until mid-exponential or late-exponential phase of growth. Expression values were calculated considering the amplification efficiencies of each primer pair used to amplify every target gene. *rpoD* gene was used to normalize. Error bars indicate the standard deviation among four measurements from two independent experiments.

We further investigated if the GIE492-encoded integrase and the putative genes encoding proteins of unknown function (*u1* to *u7*) are actually expressed in conditions in which active MccE492 is normally produced, i.e., in exponential and late exponential phase of growth (de Lorenzo, 1985; Orellana and Lagos, 1996). To this end, we performed qRT-PCR assays to detect and quantitate mRNA from those and other genes from the MccE492-production gene cluster. Transcript levels of each gene were normalized by the mRNA levels of *rpoD*, which were constant in both conditions, and were expressed as transcript abundance respect to 100 molecules of *rpoD* mRNA (**Figure 5**). We detected mRNA from all the assayed genes, including that coding for the integrase and the uncharacterized genes, indicating that all of them are transcribed. Expression levels varied among each gene, being *mceB* and *mceA* the most transcribed (around 1000 copies/100 *rpoD* molecules). The integrase-coding gene and *mceC* were also highly transcribed with values around 27 copies/100 *rpoD* molecules. Transcripts of the rest of the genes from MccE492 production cluster were slightly less abundant, with *mceJ* and *mceI* ranging from 7 to 17 copies/100 *rpoD* molecules, while *mceG* and *mceH* ranged from 2 to 10 copies/100 *rpoD* molecules. Similar expression levels were observed for the uncharacterized genes *u1* to *u4, u6* and *u7*. *u5* showed a particularly high abundance, ranging from 12 to 196 copies/100 *rpoD* molecules. The abundance of several transcripts decreased in the late exponential phase of growth, reaching up to a 16-fold and a ninefold reduction in the case of *u5* and *u6*, respectively. A moderate diminishment was observed for genes involved in MccE492 maturation (*mceC*, *mceJ* and *mceI*; 3.6-, 2-, and 2.6-fold, respectively), MccE492 export (*mceG* and *mceH*; 5.2- and 3.7-fold, respectively), and for *u7* (2.7-fold).

### The *asn1* Loci Are Hotspots for the Integration of Different Pathogenicity Islands in *K. pneumoniae*

Some reports have shown that different GIs from *K. pneumoniae* are located in the vicinity of an asn-tRNA gene, suggesting that these loci are hotspots for the integration of this kind of mobile elements (Koczura and Kaznowski, 2003; Lin et al., 2008; Zhang et al., 2011; Lery et al., 2014). However, there are no systematic studies regarding the extent and peculiarities of its usage as recombination sites in this species. The increasing number of assembled genomes of *K. pneumoniae* made available during the last 3 years allows a large-scale comparison to address this issue, to identify and classify asn-tDNA-associated islands, and to gain some insights about the properties of tRNA genes as integration sites. With this in mind, we analyzed and compared all the *K. pneumoniae* assembled chromosomes available at the NCBI database until February 1st, 2016 (a total of 52 chromosomes). We focused in determining the genomic context of all the asn-tRNA genes present in each strain and in identifying putative GIs integrated therein. To this end, we made multiple alignments of these regions to recognize conserved and strain-specific blocks of DNA, using the platforms Artemis, ProgressiveMauve, and MobilomeFINDER, as well as manual curation. The totality of the 52 chromosomes analyzed had four 100% identical copies of the gene coding for an asn-tRNA with the GUU anticodon, and lacked genes coding for the asn-tRNA with the alternative anticodon (AUU). Each of the four copies was found to be located in a specific and highly conserved genomic context (for details see Supplementary Table 3), the same shown for MGH78578 chromosome in **Figure 2** (*asn1* domain). After comparing a large number of strains, we concluded that this genetic structure corresponds to the virgin state of the four asn-tRNA loci of *K. pneumoniae*, that is to say, the structure shared by all the strains having no GIs integrated in any of them. Since the recombination site corresponds to the 3′ end of the asn-tDNA, its upstream context remains unaltered upon island integration, and thus is conserved among all strains.

**FIGURE 6:**
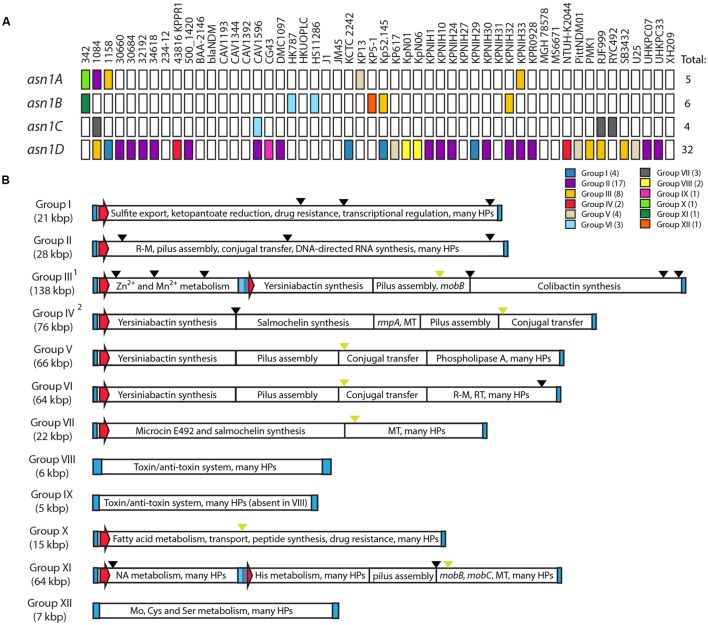
**asn-tDNA loci as integration hotspots for genomic islands in *Klebsiella pneumoniae***. **(A)** A total of 47 GIs (colored squares) integrated in any of the four asn-tRNA loci (*asn1A* to *asn1D*) were identified among the assembled chromosomes of 52 *K. pneumoniae* strains. Those GIs can be classified into 12 homology groups (see color code). **(B)** Schematic representation of the main features of each *asn1*-GI group. Blue rectangles and red arrows represent direct repeats and integrase-coding genes, respectively. Black and green arrowheads represent insertion sequences and transfer origins (oriT), respectively. ^1^Previously described as GI-I (Lery et al., 2014); ^2^Previously described as ICE*Kp1* (Lin et al., 2008). R–M, restriction–modification; HPs, hypothetical proteins of unknown function; MT, methyltransferase; NA, nucleic acids.

We identified a total of 47 GIs integrated in any of the four *asn1* loci (**Figure 6A**, Supplementary Table 4). Remarkably, 38 out of 52 strains harbor at least one *asn1*-associated GI, confirming that these loci can be considered integration hotspots. However, we noticed that GIs occupy each *asn1* locus with very different frequencies and that *asn1D* is largely preferred. This observation is unexpected considering that the four identical *asn1* genes offer the very same integration site, so it would be expected that each of them should be occupied with a similar frequency. This points out that non-considered factors could affect the selection of the GI integration site.

Comparative analysis of the identified GIs allowed us to propose a classification into 12 homology groups (I to XII), differing mainly in the sets of genes carried by the island. The bias in the integration-site selection was also observed at the level of GI groups. This is true in the case of GIE492 (group VII), where in the 38 strains known to carry this island the integration occurred at *asn1C*, the less preferred locus for the integration of the other GIs (**Figure 6A**). Additionally, in 16 of the 17 strains carrying group II-GI, and in the four strains carrying a group I-GI, the integration occurred at *asn1D.* In contrast, group III-GIs showed less marked preferences for a particular locus.

### Main Features of *asn1*-Associated Genomic Islands from *K. pneumoniae*

As mentioned above, the different GI groups carry genes that are normally clustered into modules of a particular functionality (**Figure 6B**). The members of each group differed in size from 1 to 5 kbp as a product of the variable presence of insertion sequences (ISs, black arrowheads) located at different sites, sometimes causing the interruption of one or more coding sequences. In some cases ISs are located delimitating functional modules, suggesting that homolog recombination between them could be a main force driving the shaping of GIs allowing internal rearrangements, and the acquisition or loss of gene modules. Groups III and XI corresponded to composite GIs likely formed by tandem accretion, a phenomenon in which a GI is integrated next to a previously integrated island forming a composite element that can be mobilized as a whole (Pavlovic et al., 2004).

Regarding the recombination features, all groups are flanked by very conserved direct repeats that can vary in length but preserve the core 17-bp repeat defined for GIE492, except for group XII (Supplementary Figure 1). Curiously, islands from groups VIII, IX, and XII are flanked by longer repeats of up to 148, 39, and 35 bp, respectively. Also, it was observed that a very long repeat is located between the two islands that form the composite GIs from groups III and XI. This repeat is an extension toward the 3′ direction of the 20-bp repeat flanking the composite island (shown in Supplementary Figure 1), completing a total length of around 1400 bp and comprising most of the integrase coding region. The origin and meaning of these long repeats is not clear at this stage.

The search for integrase-coding genes among *asn1*-GIs indicated that they are present in most groups except for VIII, IX, and XII. Groups III and XI have two integrase-coding genes, one at the 5′ end of each of the two GIs conforming the composite island. In order to gain information about the phylogenetic relationship between the integrase proteins encoded in *asn1*-GIs, we performed a multiple alignment of a total of 49 integrase sequences from different groups and built a distance tree (Supplementary Figure 2). Multiple alignments revealed varying degrees of sequence identity when comparing integrases from the same or different groups, ranging from 48 to 100%. In general, distribution of the integrase sequences inside the tree resembled the grouping by gene modules showed in **Figure 6**. After establishing a 90% identity cut-off, seven main phylogroups were observed. A first phylogroup was conformed by integrases encoded in GIs from group II, sharing 97–100% identity among them and a maximum identity of 51% with integrases from the other six phylogroups. A second phylogroup included integrases encoded in GIs from group VII (RJF999, 1084, and RYC492), which share a 100% identity. A third phylogroup was formed by integrases from group I, all of them 99–100% identical. The only known representatives encoded in GIs from groups X (one integrase from 342 *asn1A*-GI) and XI (two integrases from 342 *asn1B* composite GI) formed three separated phylogroups, each sharing a maximum identity of 89% with the rest of the phylogroups. Finally, the last phylogroup included 22 integrases from groups III, IV, V, and VI, all of them at least 96% identical. This suggests that islands from groups III to VI could be variants of a common ancestor that loss/acquired gene modules by homolog recombination. Considering the high conservation of the recombination module among *asn1*-associated GIs, it is plausible that they also share the general mobility properties determined experimentally for GIE492. Additionally, it could be expected that excision of *asn1*-associated GIs lacking its own integrase could be catalyzed by a close-related integrase encoded in another GI located in a distinct *asn1* locus.

Several functions related with virulence and horizontal gene transfer are encoded in the *K. pneumoniae asn1* GIs. The most prevalent modules among the 12 groups are those related with the production of yersiniabactin siderophore, pilus assembly, and conjugation. Additionally, we identified genes related with colibactin, MccE492 and salmochelin production, restriction–modification, and toxin–antitoxin systems, genes related with the metabolism of divalent cations, methylation, regulation of the mucoid phenotype, and drug resistance, among others. However, it is important to point out that nearly half of the putative genes found in the identified GIs encode hypothetical proteins with unknown function. This hampers a reliable prediction of the metabolic capabilities encoded in the islands and obscure the molecular details of their impact over the host’s phenotype.

Sequence comparison to detect shared elements between *asn1*-GIs from all groups led to an unexpected and relevant finding. A region of 300–500 bp was found to be present in GIs from groups III, IV, V, VI, VII, X, and XI (**Figure 6**, green arrowheads). This region was also found in several *K. pneumoniae* plasmids and chromosomal regions that likely correspond to mobile elements (data not shown). In a previous work describing the *K. pneumoniae* integrative and conjugative element (ICE) ICE*Kp1* (here classified as an *asn1*-GI from group IV), this region was showed to comprise a ∼250-bp transfer origin (oriT) located next to several genes coding for pilus assembly (*virB* genes) and DNA processing (*mobB* and *ardC* genes) proteins. Also, it was demonstrated that this oriT and also *virB1* and *mobB* mediate the conjugal transfer of this element, and that cloning of the oriT sequence in a plasmid vector was sufficient to allow its conjugal mobilization from a host encoding the conjugation-related proteins (Lin et al., 2008). Inspection of this region and its gene context revealed differences among GI groups, with a variable presence of genes related with conjugation or of unknown function (Supplementary Figure 3A). Similarly to group IV, in groups V, VI, and XI the conserved region is located close to several genes related with pilus assembly and DNA processing. In group III, *virB* genes were kept upstream of the conserved region, while only *mobC* was found downstream, as well as an IS that possibly mediated the loss of the other DNA processing genes found in the rest of the groups. In the case of group VII (GIE492), no conjugation-related genes were found, while for group X only *ardC* was identified. Alignment of the putative oriT found in each group revealed a high degree of conservation, including regions proposed to be relevant for its function that were described for ICE*Kp1* and also for the ICE*Ec1* mobile element found in *E. coli* (Schubert et al., 2004). Among these features, we identified two conserved inverted repeats, one direct repeat and two *nic* motifs (Supplementary Figure 3B). The high conservation of the putative oriT sequence and the experimental evidence demonstrating the conjugal transfer of ICE*Kp1* strongly suggest that GIE492 and GIs from groups III, IV, V, VI, X, and XI could be mobilized by conjugal transfer.

## Discussion

The availability of an increasing number of sequenced bacterial genomes and the development of tools allowing large-scale comparative analyses revealed that the impact of horizontal gene transfer on bacterial evolution has been largely underestimated (Dobrindt et al., 2004). Currently, it is recognized as a main force directing the arising of new strains with further metabolic capabilities and thus able to adapt to changing environments. This is also true for pathogenic bacteria, where a large set of virulence-related genes are encoded in mobile elements that can be transferred among different strains and species coexisting in a particular habitat. Understanding of such genetic-transfer mechanisms is highly relevant from a public health’s point of view, since they mediate the fast development of multidrug-resistant and hypervirulent strains causing severe clinical outbreaks. Several putative virulence factors from *K. pneumoniae* are thought to be encoded in GIs, among them the determinants for production of MccE492 and salmochelin. These GIs were mainly found integrated into certain tRNA genes, being the asn-tDNAs proposed to be an integration hotspot in this species. In this work, we analyzed all the publicly available assembled chromosomes from different *K. pneumoniae* strains searching for, classifying and characterizing a large set of asn-tRNA-associated GIs, and in particular the GIE492 island carrying the MccE492-production gene cluster. We found that 38 out of 52 strains harbored at least one GI integrated in one of the four asn-tDNAs present, confirming that these loci are integration hotspots in this species. We observed that any of the copies can be used as integration site, that they clustered together in a ∼20-kbp chromosomal segment when none of them is occupied by GIs (as shown for MGH78578, **Figure 2**), and that each copy is located in a highly conserved upstream context that remains unchanged upon GIs integration allowing the individualization of each locus (here designed *asn1A* to *asn1D*). A total of 47 *asn1*-GIs were categorized into 12 homology groups (I to XII) according to the carried genes (**Figure 6**). Among them, GIE492 corresponded to group VII consisting of highly conserved ∼23-kb GIs with the typical characteristics of this kind of element, that was found in three assembled chromosomes (RYC492, 1084, and RJF999) and in 33 partially assembled genomes from a previously sequenced set of clinical isolates (Supplementary Table 2). The rest of the groups varied in size from ∼5 to ∼138 kb and encoded a plethora of virulence-related functions.

Although all the *asn1* copies were identical, the frequencies of occupation for each of them were very different. *asn1D* was largely preferred, harboring the most prevalent *asn1*-GIs (group II) encoding proteins related with restriction–modification, conjugation, and RNA synthesis. Some groups showed a marked preference for a certain locus, while others were more evenly distributed. GIE492 was integrated into *asn1C* in all the 35 strains where it was found, while group II-GIs were integrated into *asn1D* in 16 out of 17 strains. In the case of *E. coli*, it was observed that only a small subset of tRNA genes serve as integration sites (Williams, 2002, 2003; Ou et al., 2006). In this regard, Germon et al. (2007) proposed some general rules for the usage of specific tDNAs: (1) highly transcribed tDNAs or those encoding tRNAs recognizing frequently used codons tend to be occupied at lower frequency; (2) integration into polycistronic tDNAs is generally avoided; (3) the flanking sequence context of each tDNA is an important determinant, since several tDNAs have been shown to be co-transcribed with downstream genes. Thus, disruption of such transcriptional units could therefore be detrimental to the bacteria; and (4) the local DNA structure or conformation might facilitate/disfavor the integration process. These hypotheses could explain in part the unequal frequency of occupation of each identical *asn1* locus, but do not explain why a particular island group (for example GIE492) is always inserted in a particular locus (*asn1C*) that is not used by several other GI groups. This is specially inquiring considering that most of the GI homology groups described herein share the same 17-bp direct repeat found flanking GIE492 (Supplementary Figure 1), and thus might use the same integration site. These observations point out that unconsidered factors could affect the selection of the integration site.

It is generally accepted for bacterial GIs that integration site specificity is determined by the encoded integrase. Moreover, integrases clustering together would generally use a homolog tDNA insertion site, and highly related integrases could be present on GIs encoding diverse biological functions (Williams, 2002; Boyd et al., 2009). For *K. pneumoniae asn1*-GIs we observed that the encoded integrases (when present) clustered together into seven distinct phylogroups, each of them comprising proteins sharing at least a 90% identity (Supplementary Figure 2). Integrases encoded in group II-GIs conformed the most distant phylogroup, which showed identities of up to 51% with the rest of the phylogroups. Hence, GIs encoding diverging integrases were found integrated in the same recombination site. A closer relationship was observed among the other six phylogroups, with identities ranging from 74 to 89%. GIE492 (group VII) conformed a single clade, more closely related with integrases from GI groups I, X, and XI. Also, a single phylogroup was conformed by integrases encoded in GIs from groups III to VI, sharing over a 96% identity. This observation, along with the presence of shared genetic modules suggest that these latter GI groups could correspond to variants of a common ancestor. It is important to point out that integrases from composite GIs probably originated by tandem accretion were not necessarily closely related. This could be the case of group XI where the integrases encoded in the first and the second part of the composite island formed distinct phylogroups, sharing an identity of 74% that is lower than those shared with integrases encoded in GIs from other groups and integrating in a different *asn1* locus.

Regarding the main features of GIE492, besides the previously described MccE492 determinants, this island comprised an integrase-coding gene at its 5′ end, and at least seven additional ORFs (*u1* to *u7*) of unknown function that are located at the 3′ half of the island (**Figure 2**). Gene expression analyses showed that all those genes are transcribed at levels comparable to some of the already known McE492 synthesis determinants, indicating that they actually correspond to protein-coding genes (**Figure 5**). *mceA* and *mceB*, coding for MccE492 and its immunity protein were the most transcribed genes. This could be a way of compensating the poor adaptation to the translational machinery of *K. pneumoniae* observed for these genes, which registered the lowest CAI value (**Figure 1**). Some observations suggest that the GIE492-encoded ORFs of unknown function, although not necessary for the synthesis of active MccE492, could be related with its production. First, searches in DNA-sequence databases indicated that both set of genes were always found together. Second, no ISs or repeats that account for a previous recombination event (putting the two sets together) were found. Third, GC-content and codon usage bias were homogeneous through the entire island, arguing against the possibility that both halves of the island evolved separately and joined together in a more recent event. Regarding its putative function, *u1* encodes a predicted methyltransferase with a conserved domain that in some organisms has been related with the methylation of catechols, including an example from the bacteria *Myxococcus xanthus* (Nelson et al., 2007). This raise up the possibility that MccE492 and/or salmochelin-like siderophores could be further modified by methylation. In this regard, it was shown that compounds such as 3-methylcathechol and 4-methylcathechol are found in human urine and have iron-chelator properties. Moreover, these compounds were recognized by the siderophore-binding protein siderocalin in a similar fashion than enterochelin, suggesting that they also functions as siderophores (Bao et al., 2010, 2015). At this stage, no putative roles in MccE492 production could be inferred from the predicted functions of the rest of the GIE492 uncharacterized genes. In a previous study, Lai et al. (2014) described the liver abscess-associated strain *K. pneumoniae* 1084 and reported that it carries a 208-kb asn-tRNA-associated GI named KPHPI208, which consists of eight modules including a salmochelin and microcin production module. However, the definition of the virgin state of the four *asn1* loci of *K. pneumoniae* presented in this study clearly indicates that KPHPI208 from strain 1084 actually correspond to three islands instead of only one; a group II-GI integrated in *asn1A*, a group VII-GI integrated in *asn1C* (GIE492) and a group III-GI integrated in *asn1D*.

We further provided experimental evidence indicating that GIE492 excises from the chromosome, and that its occurrence can be promoted by the DNA-damaging agent mitomycin C or overexpressing the island-encoded integrase. This effect of mitomycin C has been reported for several integrative elements such as prophages, GIs, and integrons (Bellanger et al., 2008; Ahmed et al., 2012; Puymège et al., 2013). Although its nature is not clear in most of the cases, it had been related with the activation of the SOS response as consequence of DNA damage. In this regard, a direct connection between excision induction and SOS response was demonstrated for integrons of different *Vibrio* species, where a conserved LexA-binding motif was found overlapping the promoter region of the integrase gene harbored by these elements (Guerin et al., 2009). This motif mediated the induction of the integrase-gene expression resulting in the increase of the excision frequency, upon the induction of the SOS response with several DNA-damaging agents. Conversely, no LexA-binding motifs were found in the promoter region of the GIE492-encoded integrase. Moreover, a discrete increase in the excision frequency was observed upon integrase overexpression, which was significantly lower than the mitomycin C-mediated induction. These results suggest that the effect of mitomycin C over GIE492 excision may operate by a mechanism distinct than integrase expression upregulation. The high conservation of the direct repeats and integrases among most of the *asn1*-GIs suggest that several other GI groups could share the mobility properties characterized experimentally for GIE492. Also, it allows the possibility of an integrase cross-talk, where the integrase protein encoded in an *asn1*-GI can catalyze the excision/integration of another related GI, as demonstrated for PAIs from *E. coli* 536 (Hochhut et al., 2006). This isolate contains five well-characterized PAIs (PAI I_536_ to PAI V_536_), four of them flanked by direct repeats, harboring functional integrase-coding genes, and able to excise from the chromosome. Individual inactivation of the integrase-coding genes from each PAI revealed that for PAI V_536_, excision was catalyzed by its cognate integrase but also at a comparable rate by the PAI II_536_-encoded integrase. PAI II_536_ is flanked by 18-bp direct repeats and integrated into the *leuX* tRNA gene, while PAI V_536_ is flanked by 23-bp repeats and integrated into the *pheV* tRNA gene (Middendorf et al., 2004). This points out that cross-talk could work between GIs flanked by non-identical repeats and even integrated at distinct tRNA genes. Thus, integrase cross-talk among *asn1*-GIs seems to be very likely, and would permit the excision/integration of GIs from groups VIII, IX, and XII lacking its own integrase, and also of GIs which integrase coding gene was pseudogenized.

It is important to note that asn-tRNA genes were found to be 100% identical among most species of *Enterobacteriaceae* (data not shown). In addition, experimental evidence indicates that ICE*Kp1* (group IV-GI) could be transferred by conjugation from a *K. pneumoniae* donor to an *E. coli* recipient, and that the conjugated element indeed integrated in the *E. coli* chromosome, specifically at asn-tRNA genes. This raise up the possibility that GIE492 and other *asn1*-GIs from *K. pneumoniae* could be transferred to and integrated into other related species. Nevertheless, after searching the databases we observed that no other species besides *K. pneumoniae* harbored GIE492. The same was observed for GIs from groups II, IV, and VII to XII. In contrast, homologs to group I-GIs were found in *Enterobacter aerogenes* CAV1320 and *Obesumbacterium proteus* DSM2777, although they seem to be part of a larger GI; homologs to group III-GIs were found in three strains of *Enterobacter aerogenes* (G7, EA1509E, and FDAARGOS_152); a homolog of group V-GIs was found in *Enterobacter hormaechei* 05-545; and homologs of group VI-GIs were found in two strains of *E. coli* (ACN001 and ACN002). All of those GIs were integrated into an asn-tRNA gene. This indicates that at least some *asn1*-GIs can be shared among distinct enterobacteria.

Although excision and integration of GIs are in general well-documented processes, the fate of the excised element upon its circularization and thus how can it reach a new host is in general poorly understood. In some cases, island-encoded conjugation-related determinants mediate the transfer of the element, as reported for ICEs such as ICE*Kp1* (Lin et al., 2008), here classified as a GI from group IV. ICEs (first denominated conjugative transposons) share all the distinctive features with GIs, differing only in their conjugation-related properties (reviewed in Bellanger et al., 2014). Despite there is some controversy regarding if ICEs are a particular type of GIs or different elements, we observed very similar properties among GIs encoding or not conjugative capabilities. On the other hand, many GIs lack of such conjugation determinants and their allocation to a new host may involve the capture of the island inside a phage or mobilizable plasmid and the subsequent transfer of the composite element. Here, we showed that GIE492 and other classes of *K. pneumoniae asn1*-GIs harbor a putative transfer origin located in different genetic contexts, even in absence of other conjugation-related elements. In the case of ICE*Kp1*, this region was probed to be sufficient allowing the mobilization of a plasmid where it was cloned, in a background providing the functions required for conjugation (Lin et al., 2008). Thus, it is likely that these oriT-harboring GIs could be mobilized by conjugation from hosts with such characteristics.

Our results indicated that, among a previously sequenced set of liver abscess-associated *K. pneumoniae*, 24 out of 26 isolates carry GIE492. This correlation suggests that MccE492 and or salmochelin production may play a role in the development of this kind of infection. However, there are no studies regarding the putative role of MccE492 production in pathogenesis, although it is widely accepted that this trait would permit the prevalence of the producers over surrounding cells competing for the same siderophores and thus increasing their iron supply. Unfortunately, this hypothesis has not been directly addressed and there is only evidence that *Kp*RYC492 can prevail over sensitive *E. coli* strains in mixed cultures grown in different media, but not over a microcin-resistant strain (de Lorenzo et al., 1984). This effect was also observed in anaerobic conditions, which is consistent with its proposed role in natural microbial interactions. Besides its antibacterial activity, MccE492 has two peculiar properties whose biological importance in natural conditions remains unknown. First, the purified peptide induces apoptosis and even necrosis (at higher concentrations) in some human cell lines. Moreover, this effect is also observed when incubating MccE492-producing *E. coli* with sensitive human cells (Hetz et al., 2002). In addition, MccE492 forms amyloid fibers *in vivo* both in the extracellular milieu and in the cytoplasm of producing cells, which was proposed as a mechanism to regulate its bactericidal activity (Marcoleta et al., 2013b; Aguilera et al., 2016). Additional studies are required to determine if MccE492 could act as a virulence factor and how its bactericidal, pro-apoptotic, and amyloidogenic properties affect such putative role. Regarding salmochelin production, a recent study evaluated the contribution of different siderophores in hv*Kp* infection, including aerobactin, yersiniabactin, salmochelin, and enterobactin (Russo et al., 2015). They found that in contrast to aerobactin, the inability to produce enterobactin, salmochelin or yersiniabactin (individually or in combination) did not decrease the *ex vivo* growth/survival in human ascites or serum, or decreased virulence over *in vivo* infection models. Despite that these observations argue against the importance of salmochelin production in *K. pneumoniae* pathogenesis, further studies should be performed to evaluate its specific role in liver abscess development.

## Author Contributions

AM, OM, and RL conceived the work. AM, OM, and RL designed the experiments, AM, CB-P, GN, OM, and RL analyzed the data, discussed and interpreted the results. AM, CB-P, and GN conducted the experiments. AM and RL wrote the manuscript. All the authors approved the final version of the manuscript.

## Conflict of Interest Statement

The authors declare that the research was conducted in the absence of any commercial or financial relationships that could be construed as a potential conflict of interest.

## Footnotes

1 http://www.ncbi.nlm.nih.gov/genome/

2 www.kazusa.or.jp/codon

## ABBREVIATIONS

GI: genomic island
hv*Kp*: hypervirulent *Klebsiella pneumoniae*
*Kp*RYC492: *Klebsiella pneumoniae* RYC492
MccE492: microcin E492
PAI: pathogenicity island
tDNA: gene coding for a transfer RNA
