# Supplementary material for: Klebsiella pneumoniae Asparagine tDNAs Are Integration Hotspots for Different Genomic Islands Encoding Microcin E492 Production Determinants and Other Putative Virulence Factors Present in Hypervirulent Strains
Source: Front Microbiol. 2016 Jun 3;7:849. doi: 10.3389/fmicb.2016.00849 (PMC4891358; doi:10.3389/fmicb.2016.00849)
Supplement: Supplementary file 1 [file Presentation_1.PDF]

SUPPLEMENTARY MATERIAL

Klebsiella pneumoniae asparagine tDNAs are integration hotspots for different genomic islands encoding microcin E492 production determinants and other putative virulence factors present in hypervirulent strains

Andrés Marcoleta, Camilo Berríos-Pastén, Gonzalo Nuñez, Octavio Monasterio, Rosalba Lagos.

Correspondence: Rosalba Lagos: rolagos@uchile.cl
Andrés Marcoleta: amarcoleta@uchile.cl

Supplementary Table 1. Oligonucleotide primers used in this study.

Name	Sequence (5' to 3')	Relevant features
P1	GTTTATGCCGATCTGGTCGCT	Used for the detection of GIE492 excision by nested-PCR (P1-P4), and for excision frequency determinations by qPCR (P3 and P5).
P2	GAGCATCTGCAGATAGGCATC	
P3	TCGATAATATGCGCCCCGTTCA	
P4	TCGTGAGATAAACGCTGACGTTACC	
P5	AGCGTCCGGATAAGCGAAACAA	
rpoD_realFW	CGAAGCAGTTCGATTACCTGGTCA	Used for GIE492-excision frequency determinations by qPCR.
rpoD_realRV	CTTCTCAGACCACGGCTTGTTCAT	
int_FW	ATGCAGTGCCCTGATGGAGT	Used for qRT-PCR measurements of *int* gene, coding for GIE492 integrase.
int_RV	AGCTGGTTGATTTGCCCCTGAA	
mceA_FW	GCAGGAGAGACCGATCCAAATACT	Used for qRT-PCR measurements of *mceA* gene, coding for MccE492.
mceA_RV	ACAGGGATGGGGACATTTACAGGA	
mceB_FW	TTCTATCCGCAGAGAACGCGAAAC	Used for qRT-PCR measurements of *mceB* gene, coding for MccE492 immunity protein.
mceB_RV	ACCGTAACTTCCCACCAGATACAC	
mceC_FW	CAAATGTGCGTCTGGTAGAGTGGA	Used for qRT-PCR measurements of *mceC* gene, related with MccE492 maturation.
mceC_RV	CTTTTGCATTCACAGGGCGATCAG	
mceJ_FW	GACTTGCCGAACTGGTGAAAGAAC	Used for qRT-PCR measurements of *mceJ* gene, related with MccE492 maturation.
mceJ_RV	TCAACGTATCCGGTATCCCTTCCT	
mceI_FW	ATCTTGAGGCAGATACGGAAGCAC	Used for qRT-PCR measurements of *mceI* gene, related with MccE492 maturation.
mceI_RV	ACGGGCGAAGCTGAAATAAACG	
mceH_FW	AATGGTCAATGCCGGAGACAGT	Used for qRT-PCR measurements of *mceH* gene, related with MccE492 export.
mceH_RV	TAGGTCAGCATTTCCTGCGTTGAG	
mceG_FW	TGGTTCTTTCCGTGGGCAGTTT	Used for qRT-PCR measurements of *mceG* gene, related with MccE492 export.
mceG_RV	TGACTGGCTGTCATACCGGTAACT	
U1_FW	TGCCCGGAAAGGAAATCATGTC	Used for qRT-PCR measurements of *u1* gene, of unknown function.
U1_RV	AGCGAGCAGACATCACCTGAAA	
U2_FW	GCATTCACTTATGCCGGTCAGA	Used for qRT-PCR measurements of *u2* gene, of unknown function.
U2_RV	CGGGGAGATAGTACTTCCATCA	
U3_FW	TTCATGCAGTGCTTCCCAGGAT	Used for qRT-PCR measurements of *u3* gene, of unknown function.
U3_RV	GCTGAGGTCAGCCCTGATAAAA	
U4_FW	TGTTCCAGTTCCTTCCTGGCAA	Used for qRT-PCR measurements of *u4* gene, of unknown function.
U4_RV	GTACACTGATCGTGGCGTACAA	
U5_FW	GTGCCAGTTCATGCTTTTGGGT	Used for qRT-PCR measurements of *u5* gene, of unknown function.
U5_RV	AGAGCTGCGTATGCCCTAATCA	
U6_FW	TGGATGTCGTGGGTCTGTACAA	Used for qRT-PCR measurements of *u6* gene, of unknown function.
U6_RV	AGGATAACATCGCCATTGCCTC	
U7_FW	GGCTTCCAAGACCAATGCGTAT	Used for qRT-PCR measurements of *u7* gene, of unknown function.
U7_RV	TGCCAGAGCCGCTTTATCTTTC	

Supplementary Table 2. Distribution of GIE492 among previously sequenced *K. pneumoniae* isolates (modified from Struve et al., 2015).

Isolate	Run accession[1]	Country	Year	Sample source	GIE492?	*asn1* locus
8045	ERR713646	-	-	ATCC	No	-
A5054	ERR713645	-	-	Pneumonia	Yes[2]	1C
C3091	ERR713649	U.S.	-	Urinary tract	No	-
CAS683	ERR713521	U.S.	2004	Liver abscess	Yes	1C
CAS685	ERR699266	U.S.	2005	Liver abscess	Yes	1C
CAS686	ERR713523	U.S.	2005	Liver abscess	Yes	1C
CAS687	ERR699269	U.S.	2005	Liver abscess	Yes	1C
CAS688	ERR706868	U.S.	2005	Liver abscess	No	-
CAS689	ERR706869	Canada	2005	Liver abscess	No	-
CAS690	ERR713524	Canada	2005	Liver abscess	Yes	1C
CAS691	ERR713522	Canada	2005	Liver abscess	Yes	1C
CAS692	ERR706870	Canada	2006	Liver abscess	Yes[2]	1C
CAS694	ERR712623	Canada	2006	Liver abscess	Yes	1C
CAS695	ERR712624	Canada	2006	Liver abscess	Yes	1C
CAS698	ERR713641	U.S.	2006	Liver abscess	Yes	1C
CAS699	ERR712625	U.S.	2007	Liver abscess	Yes	1C
CAS701	ERR712626	U.S.	2007	Liver abscess	Yes	1C
CAS726	ERR713642	Sweeden	2008	Liver abscess	Yes	1C
CAS727	ERR713643	Norway	2008	Liver abscess	Yes	1C
CAS813	ERR713644	Denmark	2010	Liver abscess	Yes	1C
CAS905	ERR712789	Norway	2010	Liver abscess	Yes[2]	1C
CAS906	ERR712790	Norway	2010	Liver abscess	Yes[2]	1C
CAS951	ERR718793	Norway	2012	Liver abscess	Yes	1C
CAS983	ERR712791	Taiwan	1996	Liver abscess	Yes	1C
CAS984	ERR712792	Taiwan	1996	Liver abscess	Yes	1C
CAS985	ERR712793	Taiwan	1996	Liver abscess	Yes	1C
CAS986	ERR712794	Taiwan	1996	Liver abscess	Yes	1C
CAS987	ERR712899	Taiwan	1996	Liver abscess	Yes	1C
CAS988	ERR712900	Taiwan	1996	Liver abscess	Yes	1C
CAS989	ERR712901	South Africa	1996	Pneumonia	Yes	1C
CAS990	ERR712902	South Africa	1996	Pneumonia	Yes	1C
CAS991	ERR712903	South Africa	1997	Pneumonia	Yes	1C
CAS992	ERR712904	South Africa	1997	Pneumonia	Yes	1C
Sp221	ERR713648	Denmark	1990-92	Bacteremia	Yes[2]	1C
Sp29	ERR713647	Denmark	1990-92	Bacteremia	Yes[2]	1C
TG01970	ERR732775	U.S.	2006	Respiratory tract	No	-
TG04601	ERR720244	U.S.	2005	Bacteremia	No	-
TG04605	ERR720245	U.S.	2005	Bacteremia	Yes	1C
TG04609	ERR732774	U.S.	2006	Bacteremia	No	-
TG12333	ERR720246	U.S.	2006	Bacteremia	No	-
TG12336	ERR720247	U.S.	2006	Bacteremia	No	-
TG12351	ERR720248	U.S.	2006	Respiratory tract	No	-
TG12354	ERR720249	U.S.	1999	Respiratory tract	No	-
TG12357	ERR720250	U.S.	1999	Respiratory tract	No	-
TG12366	ERR720251	U.S.	1999	Respiratory tract	No	-
TG12369	ERR732499	U.S.	2000	Respiratory tract	No	-
TG12372	ERR720252	U.S.	2000	Respiratory tract	No	-
TG12378	ERR720253	U.S.	2000	Respiratory tract	No	-
TG12387	ERR720254	U.S.	2007	Bacteremia	No	-
TG21539	ERR720255	U.S.	-	-	No	-
TG21545	ERR720256	U.S.	2008	-	No	-
TG21569	ERR720257	U.S.	1990-91	-	No	-
TG21587	ERR720259	U.S.	-	-	No	-
TG21942	ERR726901	U.S.	2010	Urinary tract	No	-
TG21950	ERR727148	U.S.	2010	Urinary tract	No	-
TG21960	ERR720260	U.S.	2011	Respiratory tract	No	-
TG21974	ERR720261	U.S.	2011	Bacteremia	No	-
TG21976	ERR720262	U.S.	2011	Bacteremia	No	-
TG21980	ERR720263	U.S.	2010	Bacteremia	No	-
TG21982	ERR720264	U.S.	2010	Bacteremia	No	-
TG21984	ERR720265	U.S.	2010	Urinary tract	No	-
TG21996	ERR727149	U.S.	2011	Urinary tract	No	-
TG22026	ERR727150	U.S.	2011	Urinary tract	No	-
TG22030	ERR727151	U.S.	2011	Urinary tract	No	-
TG22672	ERR727156	U.S.	2010	Urinary tract	No	-
TG22675	ERR727152	U.S.	2010	Urinary tract	No	-
TG22706	ERR727153	U.S.	2010	Urinary tract	No	-
TG28459	ERR727154	U.S.	2011	Urinary tract	No	-
TG30739	ERR727155	U.S.	2011	Urinary tract	No	-

1: All the runs referred herein are grouped under the primary identification number PRJEB7967 from the European Nucleotide Archive.

2: Isolates previously described as lacking MccE492-production determinants.

Supplementary Table 3. Conserved gene features located near to the four asn-tRNA genes identified in *Klebsiella pneumoniae*. When no GIs are inserted in any of the *asn1* loci the genes of this region form a 20-kbp conserved chromosomal domain. Coordinates, strand, and size details were based on MGH78578 chromosome (Genbank accession NC_009648).

context	gene[1]	Coordinates	strand	size of the encoded protein	Predicted general function[2]
upstream *1A*	*mtfA*	2,654,606-2,655,406	+	266 aa	Peptidase. Anti-repressor that participates in the regulation of glucose transport
between *1A* and *1B*	*lysR*	2,655,992-2,656,687	+	231 aa	LysR family transcriptional regulator
	erfK	2,657,066-2,658,007	-	313 aa	L,D-transpeptidase involved in peptidoglycan cross-linking
	cbl	2,658,086-2,659,036	-	316 aa	Transcriptional regulator of ABC transporters involved in sulfur and cysteine metabolism
	nac	2,659,143-2,660,060	-	305 aa	Transcriptional regulator involved in nitrogen assimilation and response to starvation
between *1B* and *1C*	*nhaP*	2,660,560-2,662,194	-	544 aa	Sodium:proton antiporter
	tetR	2,662,785-2,663,489	+	234 aa	TetR family transcriptional regulator
	yhdH	2,663,486-2,664,463	+	325 aa	Quinone oxidoreductase
	nrp	2,664,584-2,665,516	+	310 aa	Nucleoside recognition family protein, putative transporter
	lpl	2,665,427-2,667,049	-	540 aa	Alpha/beta hydrolase related with lipid metabolism
	pmrD	2,667,322-2,667,567	+	81 aa	Polymyxin resistance protein
	amn	2,667,697-2,669,151	+	484 aa	AMP nucleoside phosphorylase involved in regulation of AMP levels
between *1C* and *1D*	*yeeO*	2,669,517-2,670,953	-	478 aa	Efflux pump. Multidrug and toxic compound extrusion
downstream *1D*	*choK*	2,671,503-2,672,606	+	367 aa	Phosphotransferase. Similar to choline kinase
	dgc	2,672,603-2,673,574	-	323 aa	Diguanylate-cyclase
	cpo	2,673,781-2,674,617	-	278 aa	Non-heme chloroperoxidase, alpha/beta hydrolase

[1]: Gene names were assigned based on the names of genes encoding highly similar proteins found in the database.
[2]: General function of each protein was predicted by searching for homologs and conserved domains using BLASTp tool.

Supplementary Table 4. Overview of asn-tDNA-associated genomic islands in *Klebsiella pneumoniae.* All the identified *asn1*-GIs are shown for each strain, indicating in which locus are inserted, to which group belong (according to the numbers and colors defined in Figure 6), the coordinates and polarity in the respective chromosome, and the size (distance between the repeats, including them).

Strain	Accession	*asn1* locus	GI Group	Strand	Start	Stop	Size (bp)
342	NC_011283.1	1A	10	-	1,899,422	1,914,275	14,854
		1B	11	-	1,817,489	1,880,912	63,424
1084	CP003785.1	1A	2	-	1,922,544	1,952,393	29,850
		1C	7	+	1,885,335	1,907,621	22,287
		1D	3	-	1,744,605	1,883,457	138,853
1158	CP006722.1	1A	3	-	1,892,114	2,029,707	137,159
		1D	1	-	1,856,298	1,875,661	19,364
30660	CP006923.1	1D	2	-	1,875,215	1,902,700	27,486
30684	CP006918.1	1D	2	-	1,876,539	1,904,024	27,486
32192	CP010361.1	1D	2	-	1,790,908	1,819,593	28,686
34618	CP010392.1	1D	2	-	1,790,166	1,817,651	27,486
43816 KPPR1	CP009208.1	1D	4	-	5,241,556	5,317,763	76,208
500_1420	CP011980.1	1D	2	-	1,771,832	1,799,317	27,486
CAV1596	CP011647.1	1C	6	-	4,615,060	4,680,808	65,749
		1D	2	+	4,682,678	4,711,363	28,686
CG43	NC_022566.1	1D	9	+	2,582,915	2,587,558	4,644
DMC1097	CP011976.1	1D	2	-	1,845,363	1,872,848	27,486
HK787	CP006738.1	1B	6	-	1,801,677	1,866,227	62,186
HS11286	NC_016845.1	1B	6	+	3,433,540	3,495,709	62,170
KCTC2242	NC_017540.1	1D	1	+	3,160,180	3,179,541	19,362
KP13	CP003999	1A	5	-	1,831,298	1,893,066	61,769
KP5-1	CP008700.1	1B	12	+	391,681	398,426	6,746
Kp52.145	FO834906.1	1B	3	+	3,678,598	3,813,276	134,679
		1D	1	+	3,824,012	3,843,374	19,363
KP617	CP012753.1	1D	5	-	58,399	124,625	66,227
KpN01	CP012987.1	1D	8	-	4,582,270	4,588,371	6,102
KpN06	CP012992.1	1D	8	-	4,574,955	4,581,098	6,144
KPNIH1	CP008827.1	1D	2	+	3,467,480	3,494,965	27,486
KPNIH10	CP007727.1	1D	2	+	3,468,683	3,496,168	27,486
KPNIH24	CP008797.1	1D	2	-	2,326,326	2,353,811	27,486
KPNIH29	CP009863.1	1D	1	+	3,449,384	3,471,691	22,308
KPNIH30	CP009872.1	1D	2	+	3,457,613	3,485,098	27,486
KPNIH32	CP009775.1	1B	3	+	3,429,929	3,562,953	133,026
		1D	2	+	3,573,682	3,602,367	28,686
KPNIH33	CP009771.1	1A	3	+	3,473,517	3,611,170	137,654
		1D	2	+	3,627,974	3,655,459	27,486
KPR0928	CP008831.1	1D	2	+	3,459,009	3,487,694	28,686
NTUH-K2044	NC_012731.1	1D	4	+	3,395,820	3,472,030	76,211
PittNDM01	CP006798.1	1D	5	-	80,212	146,438	66,227
PMK1	CP008929.1	1D	3	+	5,159,087	5,262,107	103,021
RJF999	CP014010.1	1C	7	-	3,446,488	3,468,786	22,299
		1D	3	+	3,470,664	3,608,389	137,726
RYC492	APGM01000001.1	1C	7	+	1,705,122	1,727,416	22,295
SB3432	NC_021232.1	1D	3	+	3,488,043	3,525,607	37,565*
U25	CP012043.1	1D	5	+	3,705,864	3,772,089	66,226
UHKPC07	CP011985.1	1D	2	-	1,793,384	1,820,869	27,486
UHKPC33	CP011989.1	1D	2	-	1,792,161	1,819,646	27,486

(*****): This genomic island lacks the second portion aquired by tandem accretion that is present in the rest of the members of this group.

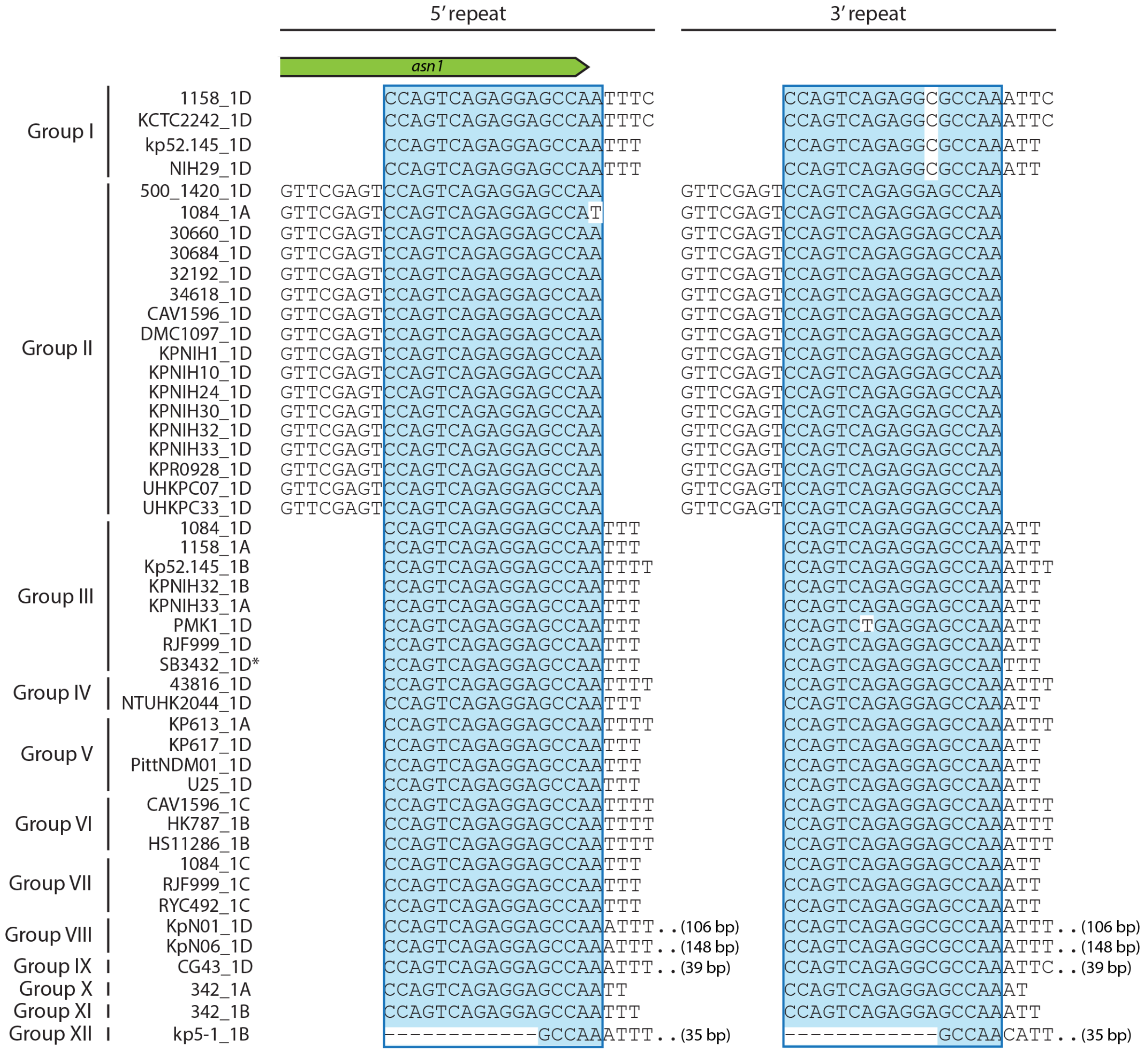

Supplementary Figure 1. Sequence alignment of the direct repeats flanking the identified asn-tDNA-associated genomic islands from *Klebsiella pneumoniae.* Repeats comprising the last sixteen bases of *asn1* flanking both the 5' and the 3' ends of each genomic island are shown. Distinct GI groups show repeats of different lenghts but almost invariably comprise the 17-bp perfect repeat described for GIE492 (shaded in blue). GIs from group VIII and IX have longer repeats of up to 148 and 39 bp, respectively. *asn1B*-GI from kp5-1 (Group XII) is flanked by more dissimilar repeats of 35-bp lenght which comprise only the last five bases of the 17-bp perfect repeat.

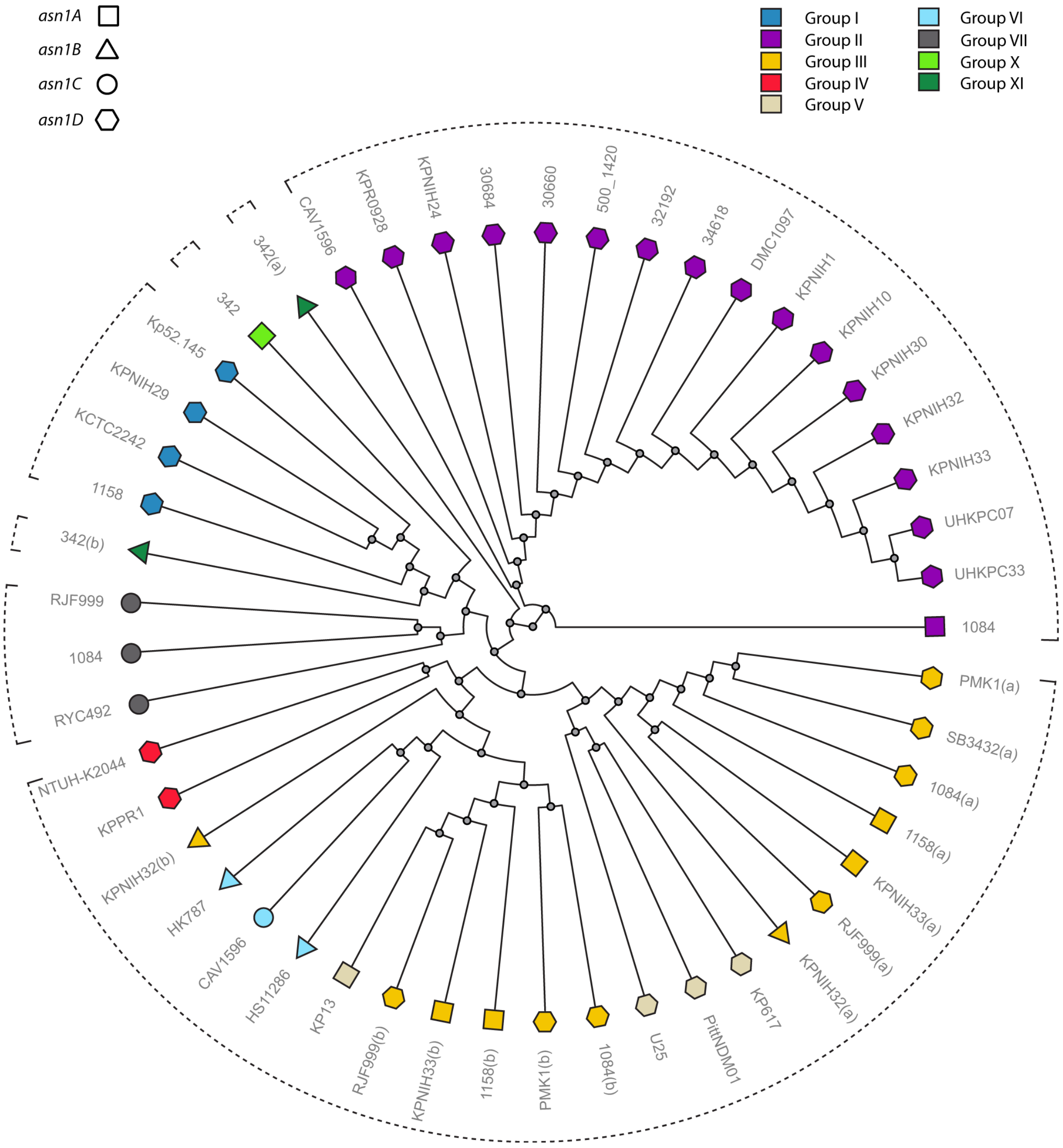

Supplementary Figure 2. Phylogenetic analysis of the integrase proteins encoded in asn-tRNA-associated GIs from *K. pneumoniae.* 49 integrase proteins from groups I to VII, X and XI were aligned generating a distance tree, using the BLAST Tree View tool and the Fast Minimum Evolution algorithm. One of four geometrical forms were assigned to each GI indicating in which *asn1* loci is integrated. The color of the form denotes the GI group. Islands from groups III and XI (except for SB3432) comprise two integrase-coding genes as result of tandem accretion, which were designated (a) or (b). Integrases from kp52.145 *asn1B*-GI (Group III) were not included since their coding genes are both pseudogenized. Groups VIII, IX and XII lack an integrase-coding gene. Dashed lines denote phylogroups which members share more than 90% identity.

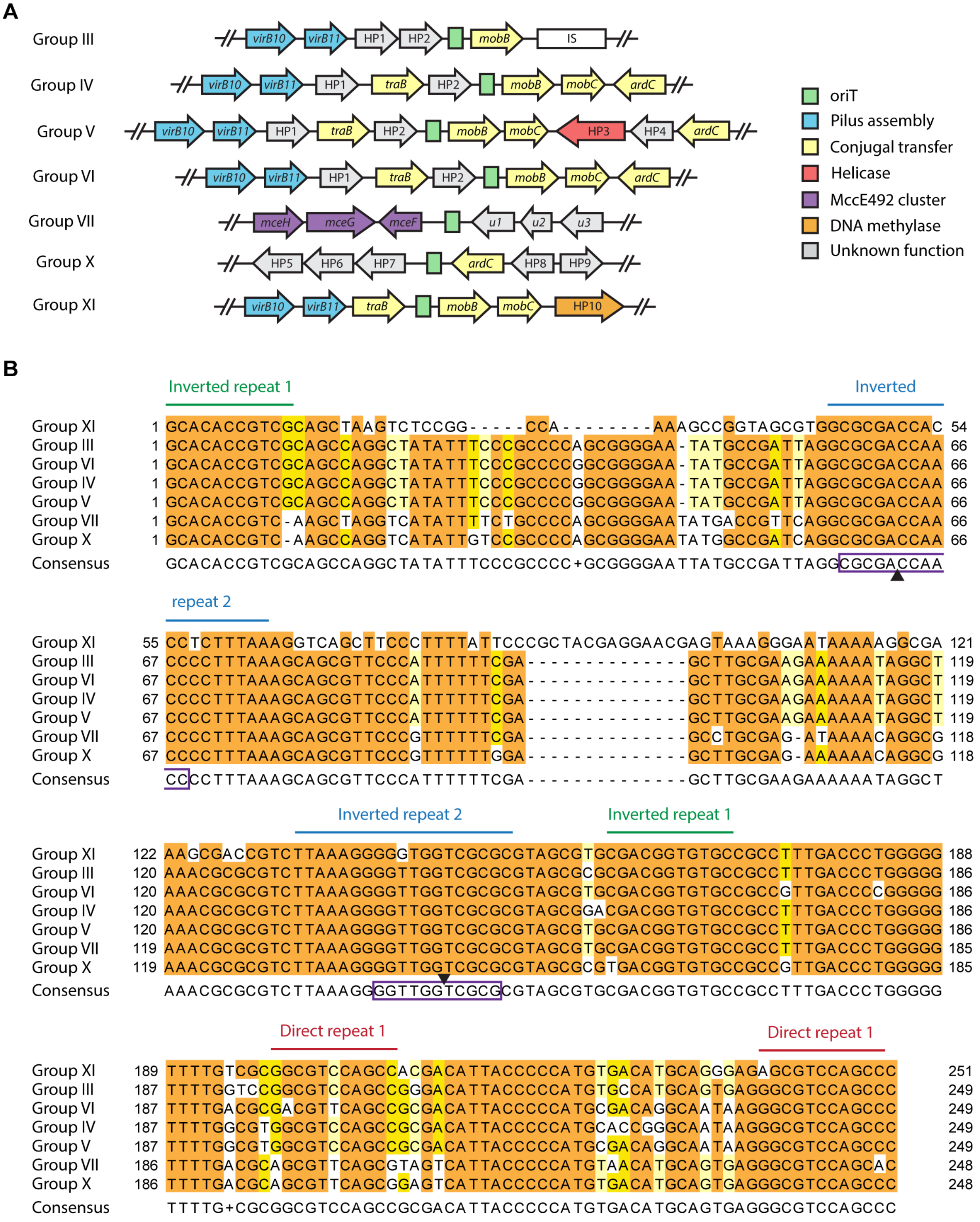

Supplementary Figure 3. A transfer origin (oriT) was identified in some groups of *asn1*-GIs from *K. pneumoniae.* (A) Gene context of the oriT identified in each GI group. (B) Sequence alignment of the ~250-bp DNA fragment comprising the oriT. Two inverted repeats and one direct repeat were found, as reported previously for one GI from group IV (ICE*Kp1*; Lin et al., 2008). Black arrowheads show the putative *nic* site, located inside the conserved nic motif (purple boxes). The position of the arrowhead indicates the strand in which the nic is generated (in top of the box: direct strand; at bottom of the box: complementary strand).
